# Efficacy and safety of tenecteplase versus urokinase in the treatment of acute ischemic stroke

**DOI:** 10.1097/MD.0000000000047879

**Published:** 2026-03-13

**Authors:** Dongxu Li, Shaoyue Huang, Xiaoxuan Wang, Zhen Hong, Xiaowei Qiu

**Affiliations:** a The Second Department of Cerebrovascular Disease, Cangzhou Central Hospital, Cangzhou City, Hebei Province, China; b Department of Neurovascular Intervention, Cangzhou Central Hospital, Cangzhou City, Hebei Province, China; c Department of Neurophysiology, Cangzhou Central Hospital, Cangzhou City, Hebei Province, China.

**Keywords:** acute ischemic stroke, intracranial hemorrhage, intravenous thrombolysis, modified Rankin Scale, National Institutes of Health Stroke Scale, recanalization, tenecteplase, urokinase

## Abstract

Acute ischemic stroke is a time-critical condition in which timely and effective intravenous thrombolysis is essential for improving neurological recovery and long-term functional outcomes. This retrospective observational study compared the clinical efficacy and safety of tenecteplase and urokinase in consecutive patients with acute ischemic stroke treated between January 2023 and December 2024. Patients were allocated to a Tenecteplase group (n = 78) or a Urokinase group (n = 82) according to the thrombolytic agent administered. Baseline demographic, vascular risk, clinical, and imaging characteristics were well balanced between groups. Early outcomes included vessel recanalization, 24-hour change in National Institutes of Health Stroke Scale (NIHSS) score, and early neurological improvement (NIHSS decrease ≥4 points). Functional outcome at 90 days was assessed using the modified Rankin Scale, while safety endpoints comprised intracranial hemorrhage (ICH), extracranial bleeding, serious adverse events, and mortality. Tenecteplase was associated with a significantly higher recanalization rate (59.0% vs 41.5%), greater 24-hour NIHSS improvement (mean ΔNIHSS 4.8 vs 3.2), and a higher proportion of patients achieving good functional outcome at 90 days (modified Rankin Scale 0–2: 84.6% vs 67.1%) compared with urokinase. Rates of ICH, symptomatic ICH, extracranial bleeding, serious adverse events, and in-hospital and 90-day mortality did not differ significantly between groups. These findings suggest that tenecteplase provides superior clinical benefit over urokinase without compromising safety in eligible patients with acute ischemic stroke.

## 1. Introduction

Acute ischemic stroke (AIS) remains a leading cause of death and long-term disability worldwide and continues to impose substantial clinical and economic burden despite advances in prevention and acute care. Early restoration of cerebral blood flow through reperfusion therapies is central to limiting infarct size and improving functional recovery. Intravenous thrombolysis has been the backbone of pharmacologic reperfusion for >3 decades, with extensive randomized evidence supporting its use in appropriately selected patients within a restricted time window after symptom onset.^[1,2]^ However, suboptimal recanalization in large-vessel occlusion, treatment delays related to infusion protocols, and bleeding risk have driven interest in alternative thrombolytic agents with more favorable pharmacological profiles. Tenecteplase is a genetically engineered variant of alteplase with increased fibrin specificity, greater resistance to plasminogen activator inhibitor-1, and a prolonged plasma half-life, which allows administration as a single intravenous bolus.^[3]^ These properties may enhance local thrombus dissolution while limiting systemic fibrinolysis and may simplify workflow in time-criticalstroke pathways. Recent large randomized clinical trials and meta-analyses have shown that tenecteplase is non-inferior to alteplase for AIS treated within 4.5 hours, with similar or higher proportions of patients achieving excellent functional outcome and no excess in symptomatic intracranial hemorrhage (ICH) or mortality.^[4]^ Network meta-analyses further suggest that tenecteplase 0.25 mg/kg ranks favorably among available intravenous thrombolytics in terms of efficacy, while maintaining an acceptable safety profile.^[5]^ On this basis, recent guidelines and expert reviews have increasingly considered tenecteplase a suitable or preferred alternative to alteplase in selected AIS populations.^[6]^

Urokinase, a plasminogen activator with lower fibrin specificity, has been used for decades as an intravenous thrombolytic agent in AIS, particularly in countries where alteplase or tenecteplase are not universally available or are cost-prohibitive. Contemporary data suggest that intravenous urokinase can achieve meaningful recanalization and functional benefits, especially when administered early, although reported outcomes vary across studies and dosing regimens. Systematic reviews and narrative overviews of novel and established thrombolytics have highlighted urokinase as a lower-cost option that may expand access to reperfusion therapy in resource-limited settings, while underscoring the paucity of high-quality comparative data with newer agents.^[7]^ In parallel, recombinant human prourokinase (another urokinase-type plasminogen activator) has demonstrated non-inferior efficacy and safety compared with alteplase in a recent randomized trial, supporting the broader concept that urokinase-class agents can be effective alternatives to rt-PA-based thrombolysis.^[8]^ Ongoing observational and interventional studies continue to explore the role of urokinase in modern stroke systems of care.

Despite these developments, direct head-to-head data comparing tenecteplase and urokinase for intravenous thrombolysis in AIS are extremely limited. Existing evidence largely derives from indirect comparisons through network meta-analyses or from single-arm urokinase cohorts, which do not fully account for differences in case mix, imaging selection, or supportive care. Given the increasing adoption of tenecteplase and the continued use of urokinase in many centers, a comparative evaluation of their real-world performance is clinically relevant. In particular, understanding how these agents differ with respect to early vessel recanalization, short-term neurological improvement, 90-day functional outcome, and hemorrhagic and nonhemorrhagic complications is essential for optimizing thrombolytic choice in diverse health-care settings. The present study was designed to address this evidence gap by analyzing a contemporary cohort of patients with AIS treated with either tenecteplase or urokinase in routine practice. By systematically comparing early recanalization and neurological outcomes, 90-day disability, and key safety endpoints, this work aims to provide clinically applicable data to inform selection of intravenous thrombolytic regimens in acute ischemic stroke.

## 2. Methods

### 2.1. Study design

This study was approved by the Ethics Committee of Cangzhou Central Hospital. This retrospective observational study included consecutive patients diagnosed with acute ischemic stroke between January 2023 and December 2024. Patients were stratified into a Tenecteplase group or a Urokinase group according to the thrombolytic regimen administered during hospitalization. The study protocol adhered to the ethical principles outlined in the Declaration of Helsinki and received approval from the institutional medical ethics committee. Written informed consent was obtained from all participants or their legally authorized representatives prior to data collection and analysis. To minimize potential selection bias, baseline demographic, clinical, and imaging characteristics were carefully compared between treatment groups. Although propensity score matching or multivariable adjustment was not performed due to the relatively balanced baseline characteristics and moderate sample size, standardized institutional thrombolysis protocols helped reduce treatment variability.

### 2.2. Inclusion and exclusion criteria

Patients were eligible for inclusion if they met the following criteria: age ≥18 years; a confirmed diagnosis of acute ischemic stroke based on clinical presentation and neuroimaging findings; symptom onset within the approved therapeutic time window for intravenous thrombolysis; and receipt of either tenecteplase or urokinase as the initial thrombolytic therapy during hospitalization. Patients were excluded if they met any of the following conditions: evidence of ICH on baseline imaging; history of hemorrhagic stroke, intracranial neoplasm, arteriovenous malformation, or aneurysm; severe coagulation abnormalities or active major bleeding; recent major surgery or trauma within the preceding 3 months; known contraindications to thrombolytic therapy, including uncontrolled hypertension or suspected aortic dissection; incomplete clinical or imaging data; or receipt of endovascular thrombectomy prior to intravenous thrombolysis.

### 2.3. Treatment protocols

Patients in the Tenecteplase group received intravenous tenecteplase in accordance with contemporary guideline-recommendeddosing for acute ischemic stroke. Tenecteplase was administered as a single intravenous bolus at a dose of 0.25 mg/kg (maximum 25 mg). The bolus was delivered over 5 to 10 seconds, followed by standard post-thrombolysis monitoring. Blood pressure, neurological status, and potential complications were assessed at predefined intervals, and emergent neuroimaging was performed if neurological deterioration occurred.

Patients in the Urokinase group received intravenous urokinase according to institutional protocols for reperfusion therapy. Urokinase was administered at a total dose of 1.0 to 1.5 million units, delivered via continuous intravenous infusion over 30 to 60 minutes. During infusion, vital signs and neurologic function were closely monitored, and infusion was discontinued immediately if signs of hemorrhage or severe adverse reactions developed. Both groups received standard supportive care, including antiplatelet or anticoagulant therapy (initiated only after post-thrombolysis imaging excluded hemorrhage), blood pressure management, glucose control, and secondary prevention measures following established clinical guidelines.

### 2.4. Data collection

Clinical data were retrospectively extracted from the institutional electronic medical record system for all consecutive patients with acute ischemic stroke who received intravenous thrombolysis with tenecteplase or urokinase between January 2023 and December 2024. Eligible cases were identified through the stroke registry and cross-checked with pharmacy records for thrombolytic use. A standardized case report form was used to collect baseline variables, including age, sex, pre-stroke modified Rankin Scale (mRS), vascular risk factors (hypertension, diabetes mellitus, atrial fibrillation, ischemic heart disease, prior stroke or transient ischemic attack, hyperlipidemia, and smoking), and prior antiplatelet or anticoagulant therapy.

Baseline clinical data included admission National Institutes of Health Stroke Scale (NIHSS) score, blood pressure, heart rate, admission blood glucose, and routine laboratory parameters when available. Time metrics (onset or last-known-well time, door-to-needle time, and onset-to-needle time) and treatment details (type and dose of thrombolytic agent, use of post-thrombolysis antithrombotic therapy, and performance of mechanical thrombectomy) were recorded. Neuroimaging data were obtained from the picture archiving and communication system. Baseline non-contrast computed tomography (CT) (and, when available, computed tomography angiography/magnetic resonance angiography) were reviewed for Alberta Stroke Program Early CT Score, presence and location of large vessel occlusion, and infarct territory. Follow-up CT or magnetic resonance imaging at 24 to 36 hours, or earlier if neurological deterioration occurred, was used to evaluate ICH and vessel recanalization. Early neurological outcomes were assessed using NIHSS at 24 hours, from which ΔNIHSS was calculated and early neurological improvement (NIHSS decrease ≥4 points) was determined. Safety events during hospitalization (any ICH, symptomatic ICH per European Cooperative Acute Stroke Study III [ECASS-III], extracranial bleeding, serious adverse events, and in-hospital death) were systematically recorded.

### 2.5. Follow-up

Patients were followed for 90 days after stroke onset. Functional outcome was assessed at 90 days using the modified Rankin Scale via outpatient clinic visits or structured telephone interviews with patients or primary caregivers. Good outcome was defined as mRS 0 to 2 and poor outcome as mRS 3 to 6. Ninety-day all-cause mortality was determined from hospital records, regional health information systems, or family reports. For patients who could not be contacted despite repeated attempts, 90-day functional status and survival were classified as missing and excluded from the corresponding analyses.

### 2.6. Statistical analysis

All statistical analyses were performed using IBM SPSS Statistics, version 26.0 (IBM Corp., Armonk). Continuous variables were assessed for normality and reported as mean ± standard deviation. Between-group comparisons were conducted using independent-samples *t* tests. Categorical variables were summarized as frequencies and percentages, and compared using χ^2^ tests without continuity correction. Early neurological outcomes were evaluated using the 24-hour change in NIHSS score (ΔNIHSS) and the proportion of patients with early neurological improvement (NIHSS decrease ≥4 points). Functional outcomes at 90 days were analyzed using both the full mRS distribution and dichotomized categories (mRS 0–2 vs 3–6). Safety outcomes (including any ICH, symptomatic ICH [ECASS-III definition], extracranial bleeding, serious adverse events, and mortality) were compared using χ^2^ tests. All statistical tests were two-sided, with a significance threshold set at *P* <.05.

## 3. Results

### 3.1. Baseline characteristics

Baseline demographic, clinical, and imaging characteristics were broadly comparable between the Tenecteplase group (n = 78) and the Urokinase group (n = 82) (Table 1). The mean age did not differ significantly between groups (67.9 ± 8.0 vs 68.7 ± 9.4 years; *t* = −0.54, *P* = .589), and the proportion of male patients was almost identical (65.4% vs 65.9%; χ^2^ = 0.00, *P* = .950). The prevalence of major vascular risk factors was similarly distributed: hypertension was present in 57.7% of patients in the Tenecteplase group and 62.2% in the Urokinase group (χ^2^ = 0.34, *P* = .561); diabetes mellitus in 17.9% versus 24.4% (χ^2^ = 0.99, *P* = .319); and atrial fibrillation in 30.8% versus 37.8% (χ^2^ = 0.88, *P* = .349), respectively. Prior antiplatelet therapy was recorded in 38.5% of patients treated with tenecteplase and 46.3% of those treated with urokinase, with no significant difference between groups (χ^2^ = 1.02, *P* = .314). Baseline clinical status was also well balanced. The mean NIHSS score at presentation was 11.5 ± 4.1 in the Tenecteplase group and 12.2 ± 4.0 in the Urokinase group (*t* = −1.11, *P* = .267). Admission systolic blood pressure was comparable (160.9 ± 16.9 vs 159.0 ± 20.4 mm Hg; *t* = 0.65, *P* = .517), as were admission blood glucose levels (both 7.9 mmol/L on average; 7.9 ± 2.0 versus 7.9 ± 1.8 mmol/L; *t* = 0.09, *P* = .926). The onset-to-needle time showed a nonsignificant trend toward longer treatment delay in the Urokinase group (173.3 ± 39.9 vs 183.3 ± 38.2 minutes; *t* = −1.62, *P* = .107). Similarly, baseline imaging parameters did not differ significantly between groups. The mean Alberta Stroke Program Early CT Score was 8.7 ± 0.9 in the Tenecteplase group and 8.9 ± 0.9 in the Urokinase group (*t* = −1.56, *P* = .122). Posterior circulation infarcts were observed in 16.7% of tenecteplase-treated patients and 25.6% of urokinase-treated patients (χ^2^ = 1.91, *P* = .167), while large vessel occlusion was present in 48.7% and 41.5% of patients, respectively (χ^2^ = 0.85, *P* = .357). Overall, no statistically significant differences were identified in baseline demographic, vascular risk, clinical, or imaging characteristics between the 2 treatment groups.

**Table 1:** Baseline demographic, clinical, and imaging characteristics of the study population.

Variable	Tenecteplase group (n = 78)	Urokinase group (n = 82)	Test statistic	*P*-value
*Demographic characteristics*				
Age (yr)	67.9 ± 8.0	68.7 ± 9.4	*t* = −0.54	.589
Male sex	51 (65.4%)	54 (65.9%)	χ^2^ = 0.00	.950
*Vascular risk factors*				
Hypertension	45 (57.7%)	51 (62.2%)	χ^2^ = 0.34	.561
Diabetes mellitus	14 (17.9%)	20 (24.4%)	χ^2^ = 0.99	.319
Atrial fibrillation	24 (30.8%)	31 (37.8%)	χ^2^ = 0.88	.349
Prior antiplatelet therapy	30 (38.5%)	38 (46.3%)	χ^2^ = 1.02	.314
*Baseline clinical status*				
NIHSS score at baseline	11.5 ± 4.1	12.2 ± 4.0	*t* = −1.11	.267
Systolic blood pressure (mm Hg)	160.9 ± 16.9	159.0 ± 20.4	*t* = 0.65	.517
Admission glucose (mmol/L)	7.9 ± 2.0	7.9 ± 1.8	*t* = 0.09	.926
Onset-to-needle time (min)	173.3 ± 39.9	183.3 ± 38.2	*t* = −1.62	.107
*Baseline imaging parameters*				
ASPECTS	8.7 ± 0.9	8.9 ± 0.9	*t* = −1.56	.122
Posterior circulation infarct	13 (16.7%)	21 (25.6%)	χ^2^ = 1.91	.167
Large vessel occlusion	38 (48.7%)	34 (41.5%)	χ^2^ = 0.85	.357

ASPECTS = Alberta Stroke Program Early CT Score, NIHSS = National Institutes of Health Stroke Scale.

### 3.2. Recanalization and early neurological outcomes

Early reperfusion and neurological recovery favored tenecteplase over urokinase (Table 2). Successful recanalization occurred in 46 of 78 patients (59.0%) in the Tenecteplase group compared with 34 of 82 patients (41.5%) in the Urokinase group, representing a statistically significant between-group difference (χ^2^ = 4.903, *P* = .027). Consistently, neurological improvement within 24 hours was greater in patients treated with tenecteplase. The mean 24-hour NIHSS change (ΔNIHSS) was 4.8 ± 3.1 points in the Tenecteplase group versus 3.2 ± 2.9 points in the Urokinase group (*t* = 3.367, *P* = .001). Early neurological improvement defined as *a* ≥ 4-point NIHSS reduction at 24 hours was observed in 56.4% of tenecteplase-treated patients and 40.2% of urokinase-treated patients, indicating a significant advantage for tenecteplase (χ^2^ = 4.185, *P* = .041). Overall, tenecteplase was associated with higher early recanalization rates and more pronounced short-term neurological recovery.

**Table 2:** Recanalization and early neurological outcomes.

Outcome	Tenecteplase group (n = 78)	Urokinase group (n = 82)	Test statistic	*P*-value
Successful recanalization on CTA/MRA/DSA, n (%)	46 (59.0%)	34 (41.5%)	χ^2^ = 4.903	.027
ΔNIHSS at 24 h (baseline − 24 h), mean ± SD	4.8 ± 3.1	3.2 ± 2.9	*t* = 3.367	.001
Early neurological improvement (NIHSS decrease ≥4 at 24 h), n (%)	44 (56.4%)	33 (40.2%)	χ^2^ = 4.185	.041

CTA = computed tomography angiography, DSA = digital subtraction angiography, MRA = magnetic resonance angiography, NIHSS = National Institutes of Health Stroke Scale, SD = standard deviation, ΔNIHSS = change in NIHSS score within 24 hours.

### 3.3. Functional outcomes at 90 days

Functional outcomes at 90 days differed between the 2 treatment groups (Table 3). The overall distribution of mRS scores showed a significant shift toward better recovery in the Tenecteplase group compared with the Urokinase group (χ^2^ = 14.051, *P* = .029). Specifically, mRS 0 was achieved in 48.7% of patients treated with tenecteplase versus 24.4% of those treated with urokinase, while death (mRS 6) occurred in 5.1% and 13.4% of patients, respectively. When outcomes were dichotomized, good functional prognosis (mRS 0–2) was observed in 66 of 78 patients (84.6%) in the Tenecteplase group and 55 of 82 patients (67.1%) in the Urokinase group, indicating a statistically significant advantage for tenecteplase (χ^2^ = 6.673, *P* = .010). Conversely, poor outcomes (mRS 3–6) were less frequent in the Tenecteplase group than in the Urokinase group (15.4% vs 32.9%; χ^2^ = 6.673, *P* = .010). Overall, tenecteplase was associated with improved 90-day functional recovery compared with urokinase.

**Table 3:** Ninety-day functional outcomes (mRS distribution and dichotomized prognosis).

Outcome	Tenecteplase group (n = 78)	Urokinase group (n = 82)	Test statistic	*P*-value
mRS score at 90 days, n (%)			χ^2^ = 14.051	.029
0	38 (48.7%)	20 (24.4%)		
1	11 (14.1%)	18 (22.0%)		
2	17 (21.8%)	17 (20.7%)		
3	3 (3.8%)	3 (3.7%)		
4	2 (2.6%)	6 (7.3%)		
5	3 (3.8%)	7 (8.5%)		
6 (death)	4 (5.1%)	11 (13.4%)		
Good functional outcome (mRS 0–2), n (%)	66 (84.6%)	55 (67.1%)	χ^2^ = 6.673	.010
Poor functional outcome (mRS 3–6), n (%)	12 (15.4%)	27 (32.9%)	χ^2^ = 6.673	.010

mRS = modified Rankin Scale.

### 3.4. Safety outcomes

Safety outcomes are summarized in Table 4. Any ICH within 24 to 36 hours was observed in 7 of 78 patients (9.0%) in the Tenecteplase group and 14 of 82 patients (17.1%) in the Urokinase group; this difference did not reach statistical significance (χ^2^ = 2.300, *P* = .129). Symptomatic ICH defined by ECASS-III criteria occurred in 2.6% of tenecteplase-treated patients and 7.3% of urokinase-treated patients (χ^2^ = 1.901, *P* = .168). Extracranial bleeding events were infrequent and comparable between groups. Any extracranial bleeding was documented in 11.5% of the Tenecteplase group versus 15.9% of the Urokinase group (χ^2^ = 0.628, *P* = .428), including gastrointestinal bleeding (3.8% vs 6.1%; χ^2^ = 0.427, *P* = .514) and subcutaneous/minor bleeding (7.7% vs 9.8%; χ^2^ = 0.213, *P* = .644). Serious adverse events during hospitalization occurred in 8 patients (10.3%) receiving tenecteplase and 15 patients (18.3%) receiving urokinase, without a significant between-group difference (χ^2^ = 2.097, *P* = .148). Mortality outcomes similarly showed no statistically significant differences: in-hospital death occurred in 2.6% of the Tenecteplase group and 6.1% of the Urokinase group (χ^2^ = 1.193, *P* = .275), while 90-day all-cause mortality was 5.1% and 13.4%, respectively (χ^2^ = 3.231, *P* = .072). Overall, tenecteplase demonstrated a safety profile comparable to urokinase, with numerically lower hemorrhagic events and mortality.

**Table 4:** Safety outcomes within 36 hours and during follow-up.

Outcome	Tenecteplase group (n = 78)	Urokinase group (n = 82)	Test statistic	*P*-value
Any intracranial hemorrhage (ICH) at 24–36 h, n (%)	7 (9.0%)	14 (17.1%)	χ^2^ = 2.300	.129
Symptomatic ICH (ECASS-III), n (%)	2 (2.6%)	6 (7.3%)	χ^2^ = 1.901	.168
Any extracranial bleeding, n (%)	9 (11.5%)	13 (15.9%)	χ^2^ = 0.628	.428
Gastrointestinal bleeding, n (%)	3 (3.8%)	5 (6.1%)	χ^2^ = 0.427	.514
Subcutaneous/minor bleeding, n (%)	6 (7.7%)	8 (9.8%)	χ^2^ = 0.213	.644
Serious adverse events (SAEs) during hospitalization, n (%)	8 (10.3%)	15 (18.3%)	χ^2^ = 2.097	.148
In-hospital mortality, n (%)	2 (2.6%)	5 (6.1%)	χ^2^ = 1.193	.275
90-day all-cause mortality, n (%)	4 (5.1%)	11 (13.4%)	χ^2^ = 3.231	.072

ECASS-III = European Cooperative Acute Stroke Study III, ICH = intracranial hemorrhage, SAE = serious adverse event, sICH = symptomatic intracranial hemorrhage.

## 4. Discussion

This study compared the efficacy and safety of tenecteplase and urokinase in patients with acute ischemic stroke treated within a standard thrombolytic time window. The main findings were that tenecteplase was associated with higher early recanalization rates, greater early neurological improvement, and better 90-day functional outcomes, while maintaining a safety profile that was not significantly different from urokinase. Taken together, these results support tenecteplase as a more favorable thrombolytic option in this clinical context. The superiority of tenecteplase in early reperfusion is reflected by the significantly higher rate of successful recanalization and greater 24-hour NIHSS improvement. Early recanalization is a key determinant of tissue salvage, infarct volume, and subsequent functional recovery. More efficient clot dissolution with tenecteplase may be explained by its greater fibrin specificity and longer half-life, which sustain thrombolytic activity at the thrombus site and may reduce systemic plasminogen activation.^[7,9]^ Enhanced early neurological improvement in the tenecteplase group, both as a continuous NIHSS change and as the proportion achieving *a* ≥ 4-point reduction, is consistent with more rapid restoration of perfusion in the ischemic penumbra. These early benefits likely contribute to the favorable shift in long-term functional outcomes observed at 90 days. The 90-day mRS distribution demonstrated a clear shift toward better outcomes in patients treated with tenecteplase, with a higher proportion attaining mRS 0 and a lower proportion experiencing death. When dichotomized, good functional outcome (mRS 0–2) was significantly more frequent in the tenecteplase group, and poor outcome (mRS 3–6) was less frequent. This pattern suggests that the early reperfusion advantage of tenecteplase translates into clinically meaningful reductions in disability. Stroke recovery is highly time- and reperfusion-dependent; thus, even modest gains in early recanalization can have cumulative impact on functional status, independence, and healthcare utilization.

The safety analysis showed no statistically significant differences between tenecteplase and urokinase in terms of ICH, symptomatic ICH, extracranial bleeding, serious adverse events, or mortality. Although absolute rates of ICH and 90-day mortality were numerically lower in the tenecteplase group, the study was not powered to detect small differences in infrequent adverse events. Nevertheless, these data indicate that the efficacy advantages of tenecteplase are not accompanied by a detectable increase in hemorrhagic risk in this cohort. The similar rates of extracranial bleeding further support the safety comparability of the 2 agents. Overall, the balance between improved efficacy and preserved safety favors tenecteplase over urokinase in this setting. From a mechanistic perspective, the observed benefits of tenecteplase over urokinase are biologically plausible. Urokinase acts primarily by converting circulating plasminogen to plasmin, with relatively less fibrin specificity, which may increase systemic fibrinolysis and bleeding risk without proportionately enhancing fibrin-bound thrombus dissolution. In contrast, tenecteplase is a genetically modified variant of alteplase with increased resistance to plasminogen activator inhibitor-1, enhanced fibrin affinity, and a longer plasma half-life, enabling single-bolus administration and sustained local thrombolytic effect at the clot surface.^[6,10]^ These pharmacological characteristics may underlie the superior recanalization and functional outcomes observed in the present study.

Although few contemporary studies directly compare tenecteplase with urokinase, the present findings are consistent with accumulating evidence that tenecteplase is at least non-inferior, and in some analyses superior, to alteplase for acute ischemic stroke. A meta-analysis of randomized controlled trials published in 2023 reported that tenecteplase and alteplase achieved comparable rates of favorable 90-day functional outcomes (mRS 0–1 and 0–2), with similar rates of symptomatic ICH and mortality, supporting tenecteplase as a reasonable alternative to alteplase in routine practice.^[11]^ Another systematic review from 2023 that examined different tenecteplase doses found that the 0.25 mg/kg dose achieved favorable efficacy and safety profiles relative to alteplase, particularly in terms of early neurological improvement, recanalization, and functional independence at 90 days.^[12]^

More recent trial and guideline data have further consolidated the role of tenecteplase. The European Stroke Organisation expedited recommendation in 2023 concluded that tenecteplase 0.25 mg/kg can be used as a safe and effective alternative to alteplase 0.9 mg/kg in patients treated within 4.5 hours, and suggested favoring tenecteplase in large vessel occlusion when feasible.^[13]^ The ATTEST-2 trial and other contemporary randomized studies reported that tenecteplase 0.25 mg/kg was non-inferior to alteplase in achieving excellent functional outcomes at 90 days, with no increase in symptomatic ICH, across a broad spectrum of stroke severities and patient subgroups.^[14]^ A 2025 meta-analysis and narrative review also indicated that tenecteplase was associated with equal or higher rates of favorable functional outcomes compared with alteplase, without an excess risk of symptomatic intracerebral hemorrhage or mortality.^[15]^ The present study extends this evidence base by focusing on urokinase, an agent still used as a thrombolytic in some regions but less intensively studied in contemporary randomized trials. While prior network meta-analyses have suggested that newer fibrinolytics such as tenecteplase and reteplase may offer improved efficacy compared with older agents, including urokinase, with similar or better safety, the current data provide direct clinical comparative evidence. The pattern of higher early recanalization and better functional outcomes with tenecteplase, coupled with comparable safety, aligns with the broader literature supporting tenecteplase as a favorable thrombolytic strategy in acute ischemic stroke.

This study has several strengths. First, it evaluated clinically relevant endpoints encompassing early recanalization, short-term neurological improvement, 90-day functional outcomes, and detailed safety measures, thereby providing a comprehensive assessment of the comparative profiles of tenecteplase and urokinase. Second, the study population reflected real-world practice, with patients treated according to routine institutional protocols and standard imaging-based stroke evaluation, which supports the external validity of the findings. Third, baseline demographic, clinical, and imaging characteristics were well balanced between groups, reducing the likelihood that observed differences in outcomes were driven by major baseline imbalances. However, important limitations should be acknowledged. The retrospective, single-center design introduces potential selection bias and unmeasured confounding, and causal inferences must be made with caution. Although sample size was sufficient to detect differences in several efficacy endpoints, it may have been underpowered to detect modest differences in infrequent adverse events such as symptomatic ICH or mortality. The choice of thrombolytic agent may have been influenced by clinician preference or logistical factors that were not fully captured in the dataset. Imaging protocols and recanalization assessments were not fully standardized, which could introduce measurement variability. Finally, follow-up was limited to 90 days, and longer-term functional and cognitive outcomes were not evaluated. Prospective, multicenter randomized studies directly comparing tenecteplase and urokinase would be valuable to confirm and extend these findings. Although the sample size was sufficient to detect differences in key efficacy outcomes such as recanalization rates and early neurological improvement, the study may have been underpowered to detect modest differences in relatively infrequent adverse events, including symptomatic ICH and mortality. Formal power calculations for rare safety outcomes were not performed, and larger multicenter studies would be required to reliably assess these endpoints. Functional outcomes were assessed at 90 days, which is a standard endpoint in stroke studies; however, longer-term follow-up such as 1-year functional status and cognitive outcomes was not consistently available in this retrospective cohort. Future prospective studies with extended follow-up would help clarify the durability of treatment benefits and long-term safety profiles.

## 5. Conclusion

In this retrospective cohort of patients with acute ischemic stroke, tenecteplase achieved higher early recanalization rates, greater 24-hour neurological improvement, and superior 90-day functional outcomes compared with urokinase, while maintaining a comparable safety profile without increased hemorrhagic events or mortality. These findings support tenecteplase as a more effective intravenous thrombolytic option than urokinase in eligible patients.

## Author contributions

**Conceptualization:** Dongxu Li, Shaoyue Huang, Xiaoxuan Wang, Zhen Hong, Xiaowei Qiu.

**Data curation:** Dongxu Li, Shaoyue Huang, Xiaoxuan Wang, Zhen Hong, Xiaowei Qiu.

**Formal analysis:** Dongxu Li, Shaoyue Huang, Zhen Hong, Xiaowei Qiu.

**Funding acquisition:** Dongxu Li, Xiaowei Qiu.

**Investigation:** Xiaowei Qiu.

**Writing – original draft:** Dongxu Li, Xiaoxuan Wang, Xiaowei Qiu.

**Writing – review & editing:** Dongxu Li, Xiaoxuan Wang, Xiaowei Qiu.
